# Dry Socket After Mandibular Third Molar Extraction: Incidence and Risk Factors in a Single-Surgeon Retrospective Study

**DOI:** 10.3390/healthcare14142037

**Published:** 2026-07-08

**Authors:** Takahiro Yamashiro, Yoichiro Ogino, Tomohiro Yamada, Masafumi Moriyama

**Affiliations:** 1 Section of Oral and Maxillofacial Surgery, Division of Maxillofacial Diagnostic and Surgical Sciences, Faculty of Dental Science, Kyushu University, Fukuoka 812-8582, Japan; jamashiro@gmail.com (T.Y.); moriyama@dent.kyushu-u.ac.jp (M.M.); 2 Yamashiro Dentistry and Oral Surgery, Fukuoka 811-3218, Japan; 3 Section of Fixed Prosthodontics, Division of Oral Rehabilitation, Faculty of Dental Science, Kyushu University, Fukuoka 812-8582, Japan; 4 Department of Oral and Maxillofacial Surgery, Nagasaki University Graduate School of Biomedical Sciences, Nagasaki 852-8588, Japan; t-yamada@nagasaki-u.ac.jp

**Keywords:** dry socket, healing capacity, mandibular third molar

## Abstract

**Objectives**: This retrospective study investigated the incidence and risk factors of dry socket following mandibular third molar extraction. **Methods**: This retrospective analysis utilized clinical information and diagnostic X-ray images of patients who underwent tooth extraction by a single oral and maxillofacial surgeon between June 2020 and March 2024. Dry socket was diagnosed according to established clinical criteria. Panoramic radiographs were used to assess angulation (Winter’s classification), depth and gingival coverage. Statistical analyses were conducted to examine differences in dry socket incidence. Categorical comparisons were performed using chi-square or Fisher’s exact tests. Multivariate logistic regression was used to identify independent risk factors for dry socket. Odds ratios with 95% confidence intervals were calculated. **Results**: A total of 3033 mandibular third molars were extracted from 2384 patients (859 males and 1525 females). The overall incidence of dry socket was 2.6% (80 cases), with a significantly higher rate in female patients (3.3%) than in male patients (1.4%) (*p* < 0.01). Increased risk was observed in age ≥ 30 years compared to early 20 s (*p* < 0.01), and the use of oral contraceptives (*p* < 0.01). Multivariate logistic regression also identified these three factors as significant independent risk factors. **Conclusions**: These findings suggest that patient-related factors influencing healing capacity contribute to dry socket development. Recognizing these associations may assist clinicians in identifying high-risk cases and implementing preventive strategies to reduce postoperative complications in third molar surgery.

## 1. Introduction

Dry socket (alveolar osteitis) is one of the most common postoperative complications after tooth extraction [1,2]. Dry socket is identified when the blood clot within the extraction socket fails to form sufficiently, resulting in exposure of the alveolar bone and severe postoperative pain. Dry socket has been defined as postoperative pain localized around the alveolus that intensifies between 1 and 3 days following tooth extraction, accompanied by partial or complete loss of the intra-alveolar blood clot, and occasionally associated with halitosis [2,3]. The typical clinical presentation consists of persistent pain localized around the extraction site, which may radiate to the temples and ears. In addition, patients often report insomnia and dizziness [1,2]. Management of dry socket primarily focuses on pain control and local wound care [1,2,3]. Conventional approaches include irrigation of the socket, avoidance of curettage, and the use of analgesics or topical anesthetic dressings. Medicated materials such as eugenol-based pastes, chlorhexidine gel, or topical anesthetic gels such as lidocaine–prilocaine are commonly used to relieve symptoms. Although systemic antibiotics are not routinely recommended, agents such as amoxicillin, metronidazole, or tetracycline have been used in selected cases with signs of secondary infection. Recent adjunctive therapies, including low-level laser therapy, probiotics, and biologically active materials such as platelet-rich fibrin (PRF) or plasma rich in growth factors (PRGF), have also been reported to promote healing and reduce postoperative discomfort [1,2,3]. These management strategies highlight the clinical importance of identifying patients at increased risk of developing dry socket.

The development of dry socket has been associated with various factors [1,2,3,4,5,6,7]. Although these multiple factors have been discussed in previous reviews, the findings from previous studies were based on relatively small sample sizes [6,7,8,9,10,11], and the definitive risk factors remain controversial. The relative contribution of surgery-related versus patient-related factors remains unclear, partly because previous studies often involved multiple surgeons with heterogeneous techniques. As a result, it is difficult to determine whether dry socket is primarily driven by procedural variability or by patient-specific healing capacity. In particular, the incidence of dry socket is higher in mandibular third molar sites [2], and understanding the risk factors for dry socket is crucial to provide appropriate preoperative informed consent, exercise intraoperative caution, and deliver effective postoperative explanations and management.

This retrospective study aimed to examine the incidence of dry socket and its associated factors in mandibular third molar extraction cases performed by a single oral and maxillofacial surgeon, to minimize operator-dependent variability and to precisely identify more objective risk factors. The hypothesis was that the occurrence of dry socket after mandibular third molar extraction is differentially influenced by patient-related and procedure-related factors, and that patient-related factors may play a more substantial role when surgical procedures are performed under standardized conditions.

## 2. Materials and Methods

### 2.1. Ethics Statement

This retrospective study was approved by the institutional review board for clinical research (approval number: 23038–00). All procedures were conducted in accordance with the Declaration of Helsinki and STROBE (Strengthening the Reporting of Observational Studies in Epidemiology) guidelines. All analyzed image data were securely stored on an institutional image server and made available for academic research purposes.

### 2.2. Study Participants

This retrospective analysis utilized patient clinical information and diagnostic X-ray images. All patients who underwent mandibular third molar extraction between June 2020 and March 2024 were the potential study participants. Given the retrospective design of the study, the institutional review board approved a waiver of informed consent. Patients who met the exclusion criteria were excluded. The exclusion criteria were as follows:

- Presence of cyst-like radiolucent lesions;
- Undergoing coronectomy;
- Ectopic impaction of the mandibular third molar.

Acute inflammatory conditions such as pericoronitis were managed with appropriate medication prior to extraction, and no teeth were removed under active acute infection. Patients with systemic diseases were included only when their conditions were medically controlled and stable at the time of surgery. Because this study was retrospective in design, sample size calculation was not performed; instead, all eligible mandibular third molar extractions conducted during the study period were included.

### 2.3. Surgical Procedure

The tooth extraction was performed only after the resolution of any acute inflammation. All procedures were carried out under local anesthesia, and when necessary, mucoperiosteal flap elevation, bone removal, tooth sectioning, saline irrigation, and suturing were performed using standard oral surgical techniques by a single experienced surgeon. In accordance with the 2015 clinical practice guideline for third molar surgery issued by the Japanese Society of Chemotherapy and the Japan Society for Surgical Infection [12], all patients received a single dose of antibiotics one hour before surgery as part of the standard perioperative management. Amoxicillin (250 mg) was used as the first-line agent, and clindamycin (300 mg) was prescribed in cases of penicillin allergy.

### 2.4. Data Extraction

Patient data including sex, age, smoking status, and medication, and postoperative occurrence of dry socket were collected from medical records. Dry socket was diagnosed by the same oral and maxillofacial surgeon who performed all extractions, ensuring standardized assessment. The diagnostic criteria were based on established definitions in the literature [2] and included:

- postoperative pain occurring 2–5 days after extraction that increased in severity;
- partial or complete loss of the intra-alveolar blood clot with or without exposed bone;
- the presence of a putrid or fetid odor or halitosis when present;
- radiating pain to the ear or temporal region as supportive findings.

Topical anesthetic application to the socket provided temporary pain relief and was used as an additional clinical indicator.

To evaluate the condition of the mandibular third molars, their angulation and depth of impaction were retrieved and analyzed from panoramic radiographs. Panoramic images were acquired using the Promax^®^ 3D Plus system (Planmeca, Helsinki, Finland), and the angulation of the mandibular third molars was measured using Planmeca Romexis^®^ 4.6.2.R software (Planmeca, Helsinki, Finland).

The angulation of the mandibular third molars was categorized according to Winter’s classification [13]. Angle thresholds were defined based on previous studies as follows [14,15]:

- Vertical (−10° ≤ angle ≤ 10°);
- Mesioangular (10° < angle ≤ 80°);
- Horizontal (80° < angle ≤ 100°);
- Distoangular (−80° ≤ angle < −10°);
- Inverted (>100° or <−80°).

The impaction status of the mandibular third molars was classified as either complete or partial. Complete impaction was defined as the tooth being entirely embedded within the gingiva. Partial impaction was defined as the presence of visible crown portions through the gingival tissue. Additionally, the depth of impaction was classified as either shallow or deep. Shallow impaction was defined when the highest point of the third molar exceeded half the height of the crown of the adjacent second molar, whereas deep impaction was defined when the highest point was below this threshold.

Extracted teeth were subsequently evaluated based on the presence or absence of dental calculus on the surface. Surgical duration was obtained from medical records and dichotomized using the mean operative time of the dataset (seven m) as a data-driven threshold, allowing comparison between procedures shorter and longer than the average duration.

### 2.5. Statistical Analyses

Statistical analyses were performed to evaluate differences in dry socket incidence across categorical variables. For two-group comparisons, the chi-square test was used when all expected cell counts were ≥5, whereas Fisher’s exact test was applied when any expected count was <5. For comparisons across multiple age and angulation categories, the chi-square test was also employed.

A multivariate logistic regression analysis was conducted to identify independent risk factors associated with the occurrence of dry socket. To address the potential non-independence of observations arising from patients who contributed two mandibular third molars, we conducted two multivariate logistic regression analyses: one including all extracted teeth and another restricted to patients who contributed only a single extraction. This sensitivity analysis allowed us to evaluate whether within-patient clustering influenced the stability of the identified risk factors. Clinically relevant variables including sex, side, age (<30 years or ≥30 years), smoking status, medication (none, oral contraceptives, antidepressant, antiallergic drugs, and other medication), angulation (horizontal or other angulations), impaction (complete or others), depth, dental calculus, and surgical duration (<seven minutes or ≥seven minutes) were adapted as explanatory variables in the model, and the final set of predictors was determined using a stepwise selection procedure. A multivariate logistic regression analysis was performed using a predefined set of clinically relevant variables. The final model was determined using a stepwise selection procedure, which retained variables that significantly improved model fit. Odds ratios (ORs) with 95% confidence intervals (CIs) were calculated.

All statistical analyses were performed using the EZR software (version 1.54: Saitama Medical Center, Jichi Medical University, Saitama, Japan) and statistical significance was defined as *p* < 0.05.

## 3. Results

### 3.1. Study Participants and Analysis of Extracted Third Molars

The study participants consisted of 2384 patients, including 859 males (36.0%) and 1525 females (64.0%). Table 1 presents the distribution of extracted mandibular third molars. Briefly, a total of 3033 mandibular third molars were extracted during the study period, including 1097 teeth (36.2%) from male patients and 1936 teeth (63.8%) from female patients. Table 1 also presents the extraction side (right or left) and the distribution of age among the extracted mandibular third molars.

Table 2 presents the detailed characteristics of mandibular third molars that developed dry socket. Dry socket was diagnosed in 80 cases, resulting in an overall incidence rate of 2.6%.

Regarding sex, dry socket occurred in 15 sites in males (1.4%) and 65 sites in females (3.4%), with females showing a significantly higher incidence (*p* < 0.01, chi-square test). Dry sockets were also found in 46 sites on the right side (3.0%) and 34 on the left side (2.3%), with no significant difference between sides (Table 2).

The effect of age on the incidence of dry socket was also evaluated. Participants were categorized into five age groups, and the incidence increased from the early 20 s to the late 30 s. Chi-square analysis followed by Bonferroni-corrected post hoc comparisons revealed that participants in their early 30 s, late 30 s, and over 40 s had significantly higher incidence than those in their early 20 s (*p* < 0.01 for all comparisons) (Table 2).

The effect of smoking was also statistically analyzed. Dry socket was identified in nine of 340 sites in smokers (2.6%) and 71 of 2693 sites in non-smokers (2.6%), with no statistically significant difference (Table 2).

In terms of medications, the incident rates were calculated and statistically compared between the patients with and without medication use (Table 3). Dry socket occurred in 8 cases (15.4%) among patients taking oral contraceptives, which was significantly higher than in those not taking them. No significant differences in incidence were detected for other medications.

The effect of characteristics of mandibular third molars and surgical intervention on the incidence of dry socket was statistically analyzed (Table 4). Differences in the incidence of dry socket were analyzed in relation to the eruption characteristics of mandibular third molars, based on three parameters: angulation, impaction status, and depth of impaction. Regarding angulation, dry socket occurred in 15 cases (3.0%) with vertical impaction, 27 cases (3.2%) with mesioangular impaction, 34 cases (2.1%) with horizontal impaction, and four cases (3.4%) with distoangular and inverted impaction. No significant differences in incidence were observed among the angulation categories. The incidence rates were 2.7% for complete and 2.6% for partial impaction, and 2.7% for shallow and 2.4% for deep impaction, respectively. No significant differences in the incidence of dry socket were found with respect to impaction status (complete vs. partial) or impaction depth (shallow vs. deep).

The effect of dental calculus on the incidence of dry socket was also statistically evaluated. In the present cohort, the incidence of dry socket among cases with visible dental calculus on the crown surface of the extracted mandibular third molars was 2.3%, whereas the incidence among cases without calculus was 3.2%. No significant difference in dry socket incidence was observed between cases with and without dental calculus on the crowns of the extracted mandibular third molars.

The influence of surgical duration on dry socket incidence was also evaluated. Dry socket occurred in 49 cases (2.8%) when the surgical duration was seven minutes or longer, and in 31 cases (2.4%) when the surgical duration was less than seven minutes. This category also showed no statistical differences.

### 3.2. Risk Factors Identified Through Multivariate Analysis

Among the explanatory variables listed above, five variables were included in the final multivariate logistic regression model based on all extracted mandibular third molars (Table 5). Female sex, age ≥30 years and the use of oral contraceptives were significantly associated with an increased risk of dry socket (*p* < 0.01), whereas horizontal impaction and the presence of dental calculus were not significantly associated.

To further assess the potential influence of within-patient clustering, an additional multivariate logistic regression analysis was performed using only patients who contributed a single extraction (1735 patients) (Table 6). In this sensitivity analysis, the explanatory variables included female sex, age ≥ 30 years, extraction on the right side, and the use of oral contraceptives. The significant predictors including female sex, age ≥ 30 years, and oral contraceptive use were identical to those observed in the full dataset, indicating that the main findings were robust to the restriction to single-extraction patients.

## 4. Discussion

The primary pathogenesis of dry socket is thought to involve impaired blood supply and premature fibrinolysis [1,2,3,4,5]. Although a variety of potential risk factors have been recognized [1,2,3,4,5,6,7,8,9,10,11], these factors vary considerably across previous studies, partly due to differences in surgical technique and operator experience. In this study, all surgical procedures were performed by a single oral and maxillofacial surgeon, and this uniformity substantially reduced procedural variability and enabled a more reliable assessment of potential risk factors.

In the present study, dry socket was observed in 80 of the 3033 extracted mandibular third molars, yielding an incidence of 2.6%. Although previous reviews and studies have reported a wide range in the incidence of dry socket, from 1% to 45% [2,3,4,5,6,7,8,9,10,11], the incidence in our cohort fell within this wide range but remained on the lower side compared with earlier studies. This relatively low incidence may be attributed to the methodological characteristics of our study, particularly the single-surgeon design in which all procedures were performed by an experienced oral and maxillofacial surgeon. This ensured consistent surgical technique, minimized procedural trauma, and allowed careful postoperative bleeding control. The uniformity of the surgical procedures likely reduced operator-related variability, providing a plausible explanation for why our incidence was lower than that reported in many previous studies.

This study clearly indicated that female sex, age ≥ 30 years, and the use of oral contraceptives could be potential risk factors for dry socket. Although surgical technique, traumatic surgery, or the operator’s expertise have been suggested as potential risk factors [1,2,3,4,5,6,7,8,9,10,11], this study included only cases treated by a single experienced oral and maxillofacial surgeon. Therefore, factors related to surgical technique or operator variability may not have been detected, which could be considered both a strength and a limitation of this study.

Female sex has been reported as a potential risk factor compared to male sex [3,4,5,7,8,10,11]. However, the effect of sex remains controversial [9,16]. Several studies have suggested the influence of menstrual cycle on the incidence of dry socket [1,2,3,4,5,8,9,11,17,18]. Menstrual cycle is related to estrogen level. Higher level of female sex hormones, predominantly estrogen, is potentially associated with higher fibrinolytic activity [17,18,19,20]. Although these mechanisms are biologically plausible, they remain speculative in the context of the present study, as hormone levels, fibrinolytic markers, and menstrual-cycle phase were not measured.

Regarding age, the patients aged in their early 20 s showed a significantly lower incidence of dry socket than those over 30 years. Although previous studies have reported inconsistent findings [1,2,3,4,5], this may partly reflect differences in age-group classification. Mandibular bone marrow decreases with aging [21,22], resulting in reduced blood supply and impaired clot stability. Additionally, dry socket is generally observed more frequently in the mandible than in the maxilla [1], likely reflecting anatomical differences in bone marrow characteristics [23]. These findings support the present result that older individuals may be at higher risk of developing dry socket than younger individuals.

As the previous reports indicated [1,2,3,4,24,25,26], the present study clearly showed that the use of oral contraceptives is one of the significant risk factors for dry socket. Increased estrogen associated with oral contraceptives may enhance plasma fibrinolysis [4,18,26]. This mechanism may contribute to dry socket development, similar to the increase in estrogen level attributed to the menstrual cycle. Although some reports did not observe this effect [27,28], it may be advisable to pay particular attention to patients using oral contraceptives. However, because hormonal and fibrinolytic parameters were not assessed, this mechanism should be considered a potential explanation rather than a direct conclusion.

The present study clearly demonstrated that significant risks of dry socket were associated with patient-related factors that may influence healing capacity, including sex (female), age, and the use of oral contraceptives, rather than surgical intervention and eruption status. In this study, all procedures were performed by a single oral surgeon, and careful attention was paid to postoperative bleeding. These factors may have influenced the results. Based on this background, further discussion regarding smoking habit and dental calculus deposition should be described. Whereas smoking has been regarded as one of the risk factors of dry socket due to the impaired healing potential [1,2,3,4], the evidence remains controversial [5]. Smoking may contribute to the development of dry socket through vasoconstriction, impaired blood supply, delayed wound healing, and mechanical disruption of the blood clot [1,2,3,4,5,6,7,8,9,10,11]. In recent years, several preventive approaches—including prophylactic antibiotics, probiotics, and platelet concentrates such as PRP and PRF—have been proposed [29,30]. Although prophylactic antibiotics and PRF/PRP may reduce the incidence of dry socket, the routine use of antibiotics remains controversial due to concerns regarding antimicrobial resistance and adverse effects. In contrast, probiotics may represent a potential future strategy through modulation of the oral microbiota, although direct clinical evidence remains limited. A more detailed evaluation, including the number of cigarettes smoked, should be considered. Additionally, oral hygiene status can be considered as one of the risk factors of dry socket. Dental calculus deposition, as an indicator of oral hygiene status in this study, was not identified as a significant risk factor. Interpretation of smoking and oral-hygiene effects should also be cautious, as smoking was recorded only dichotomously and dental calculus represents an indirect and incomplete indicator of oral-hygiene status. Further studies would be favorable to evaluate oral hygiene status using some indicators such as plaque control record. A likely explanation for the absence of significant associations between local factors and dry socket is the highly standardized surgical environment in this study. Under such controlled circumstances by a single experienced surgeon, patient-related factors that inherently affect healing capacity—such as female sex, older age, and oral contraceptive use—are more likely to influence clot stability and the development of dry socket.

Recent literature has also emphasized broader aspects of third molar surgery, including updated risk-assessment guidance and the use of biologically active adjuncts to support socket healing [31,32,33]. Although these approaches differ from the focus of our study, they similarly highlight the importance of the patient’s healing capacity and biological environment, which is consistent with our finding that systemic factors outweighed local surgical variables.

This study has several limitations. First, because it was conducted retrospectively at a single institution and included only Japanese patients, potential differences related to ethnicity could not be evaluated. As with any retrospective design, the analysis was dependent on the availability and quality of existing clinical records and missing or incomplete data may have influenced the accuracy of some variables. Seasonal or climatic influences on postoperative healing were also not assessed, as such information was not available in the clinical records.

Second, although some patients contributed two mandibular third molars, bilateral extractions were analyzed at the tooth level, and potential intra-patient clustering may not have been fully accounted for. However, no multiple extractions were performed on the same day, and the anatomical and clinical conditions differed substantially between sides. In addition, all procedures were performed by a single experienced surgeon using a standardized technique. This single-surgeon design minimizes operator-dependent variability and strengthens internal consistency, but it also limits the generalizability of the findings to other clinicians or surgical approaches. Furthermore, to evaluate the potential impact of within-patient clustering, we conducted both a primary multivariate analysis including all extracted teeth (3033 teeth) and a sensitivity analysis restricted to patients with only a single extraction (1735 teeth); the significant predictors were identical in both analyses, indicating that the main findings were robust.

Third, detailed information on smoking intensity, oral hygiene status, and perioperative antibiotic use was not collected. Although all patients received a single preoperative dose of antibiotics one hour before surgery in accordance with the 2015 Japanese guideline [12], similar perioperative antibiotic regimens—including preoperative amoxicillin—have also been reported in international evidence such as a Cochrane systematic review [34]. However, because individual-level antibiotic data were not collected, the evaluation of their potential influence may have been limited.

Fourth, the threshold of seven minutes reflects the distribution of operative times in our cohort rather than a clinically established boundary and should therefore be interpreted with caution.

Fifth, this retrospective design inherently carries risks of bias. Although selection bias was minimized by including all eligible cases within the study period, information bias may still exist because smoking status and oral-hygiene indicators were recorded in simplified categories. Additionally, residual confounding cannot be fully excluded despite the inclusion of multiple clinically relevant variables in the multivariate models. These limitations should be considered when interpreting the findings. In addition, multilevel logistic regression would be ideal for accounting for the hierarchical structure of teeth nested within patients. As an alternative, we performed a sensitivity analysis restricted to single-extraction patients, and the results were consistent with the primary analysis, suggesting that the influence of clustering was limited.

Finally, although multilevel logistic regression would theoretically allow simultaneous modeling of tooth-level and patient-level effects, the retrospective nature and scale of the dataset limited the feasibility of constructing a hierarchical model. Several other tooth-level parameters required for multilevel modeling such as precise angulation measurements, three-dimensional positional relationships, periodontal status, and pre-existing bone loss, were not available in the clinical records. Therefore, while all reliably documented tooth-level variables were incorporated into the multivariate model, a full hierarchical analysis could not be performed and remains an inherent limitation of the study. To partially address the issue of patient-level clustering, we performed a sensitivity analysis restricted to patients who contributed only a single extraction. The significant predictors identified in this analysis were identical to those in the full dataset, suggesting that the influence of within-patient clustering on the overall conclusions is likely to be limited.

Despite these limitations, the study has notable strengths, including a large sample size and the methodological advantage of a single experienced surgeon performing all procedures, which minimized operator-dependent variability and enabled a more consistent assessment of patient-related risk factors. In addition, the findings of this study suggest that patient-related factors influencing healing capacity play a pivotal role in the development of dry socket. Therefore, future preventive strategies should focus on maintaining an environment that supports natural wound healing and minimizing factors that may compromise clot stability. Adjunctive approaches such as probiotics or platelet concentrates may offer additional benefits. Nevertheless, optimizing the patient’s healing capacity remains the most fundamental strategy. Although these findings may not be fully generalizable to all clinicians, they remain noteworthy and these results may contribute to improved strategies for preventing dry socket.

## 5. Conclusions

Within the limitations of this study, the present study demonstrated that patient-related factors that may influence healing capacity, including sex (female), age, and the use of oral contraceptives, were significantly associated with the incidence of dry socket after mandibular third molar extraction, whereas surgical factors and eruption status were not identified as significant factors under the procedures by a single oral surgeon. These results suggest the importance of patient characteristics in risk assessment. However, this conclusion should be interpreted with caution. Although surgical procedures were standardized in this study, unmeasured procedural nuances and perioperative variables may still influence healing outcomes.

These findings have practical implications for clinical care. Because patient-related factors played a more prominent role than surgical variables, preoperative risk assessment and tailored postoperative management may be particularly important for individuals with reduced healing capacity. This perspective may help clinicians better identify patients who require closer monitoring or preventive strategies.

## Figures and Tables

**Table 1:** Distribution of extracted mandibular third molars.

Category		Number of Mandibular Third Molars (%)
Total		3033 (100)
Sex	Male	1097 (36.2)
	Female	1936 (63.8)
Side	Right	1545 (50.9)
	Left	1488 (49.1)
Age	Early 20 s (20–24)	742 (24.4)
	Late 20 s (25–29)	567 (18.7)
	Early 30 s (30–34)	521 (17.2)
	Late 30 s (35–39)	433 (14.3)
	Over 40 s (≥40)	770 (25.4)

**Table 2:** Incidence of dry socket in mandibular third molars.

Category		Number of Mandibular Third Molars	Number of Dry Socket Teeth (%)	*p*-Value
Total		3033	80 (2.6)	
Sex	Male	1097	15 (1.4)	<0.01 *
	Female	1936	65 (3.4)	
Side	Right	1545	46 (3.0)	0.26 *
	Left	1488	34 (2.3)	
Age	Early 20 s (20–24)	742	5 (0.7)	<0.01 * vs. Early 20 s (post hoc) Early 30 s <0.01 ** Late 30 s <0.01 ** Over 40 s <0.01 **
	Late 20 s (25–29)	567	12 (2.1)	
	Early 30 s (30–34)	521	20 (3.8)	
	Late 30 s (35–39)	433	18 (4.2)	
	Over 40 s (≥40)	770	25 (3.2)	
Smoking	+	340	9 (2.6)	1.0 *
	−	2693	71 (2.6)	

* *p*-values were calculated using the chi-square test. ** Post hoc pairwise chi-square tests with Bonferroni correction.

**Table 3:** Incidence of dry socket according to medication use.

Medication	Number of Mandibular Third Molars	Number of Dry Socket Teeth (%)	*p*-Value *
None	2479	59 (2.4)	0.08 *
Oral contraceptives	52	8 (15.4)	<0.01 **
Antidepressant	65	4 (6.2)	0.20 **
Antiallergic drugs	88	2 (2.3)	1.0 **
Others	407	11 (2.7)	1.0 *

* *p*-values were calculated using the chi-square test. ** *p*-values were calculated using Fisher’s exact test.

**Table 4:** Incidence of dry socket according to status of mandibular third molars and surgical duration.

Category	Number of Mandibular Third Molars	Number of Dry Socket Teeth (%)	*p*-Value *
Angulation			
Vertical	495	15 (3.0)	0.35
Mesioangular	834	27 (3.2)	
Horizontal	1587	34 (2.1)	
Distoangular and Inverted	117	4 (3.4)	
Impaction			
Complete	1210	33 (2.7)	0.89
Partial	1823	47 (2.6)	
Depth			
Shallow	2550	68 (2.7)	0.70
Deep	483	12 (2.4)	
Dental calculus			
+	1810	41 (2.3)	0.15
-	1223	39 (3.2)	
Surgical duration			
≥seven minutes	1305	31 (2.4)	0.50
<seven minutes	1728	49 (2.8)	

* *p*-values were calculated using the chi-square test. No statistically significant differences were observed.

**Table 5:** Multivariate logistic regression analysis of risk factors for dry socket based on all extracted mandibular third molars.

Category	OR	95% CI	*p*-Value
Female	2.4	1.4–4.3	<0.01
Age ≥ 30 years	3.9	2.2–6.9	<0.01
Oral contraceptives (+)	10.8	4.6–25.9	<0.01
Horizontal	0.6	0.4–1.0	0.05
Dental calculus (+)	0.7	0.5–1.1	0.2

OR: odds ratio, CI: confidence interval.

**Table 6:** Multivariate logistic regression analysis of risk factors for dry socket in patients with a single extracted mandibular third molar.

Category	OR	95% CI	*p*-Value
Female	2.4	1.2–5.1	0.02
Right	1.6	0.9–2.9	0.1
Age ≥ 30 years	2.6	1.3–6.9	<0.01
Oral contraceptives (+)	8.6	2.9–5.1	<0.01

OR: odds ratio, CI: confidence interval.

## Data Availability

The data supporting the findings of this study are not publicly available due to privacy and ethical restrictions. De-identified data may be made available from the corresponding author upon reasonable request.

## Abbreviations

The following abbreviations are used in this manuscript:

STROBE	Strengthening the Reporting of Observational Studies in Epidemiology
OR	Odds ratio
CI	Confidence interval
